# Dapagliflozin ameliorates intestinal stem cell aging by regulating the MAPK signaling pathway in *Drosophila*

**DOI:** 10.3389/fcell.2025.1576258

**Published:** 2025-04-23

**Authors:** Jinhua Yan, Chenxi Feng, Hanmei Zhang, Ting Luo, Haiyang Chen, Haiou Chen

**Affiliations:** ^1^ Center of Gerontology and Geriatrics and Laboratory of Metabolism and Aging Frontiers Science Center for Disease-Related Molecular Network, State Key Laboratory of Respiratory Health and Multimorbidity and National Clinical Research Center for Geriatrics, West China Hospital, Sichuan University, Chengdu, China; ^2^ West China School of Medicine, Sichuan University, Chengdu, Sichuan, China

**Keywords:** dapagliflozin, intestinal stem cell, aging, MAPK signaling, *Drosophila*

## Abstract

**Introduction:**

Intestinal stem cells (ISCs) possess the ability to self-renew and differentiate, which is essential for maintaining intestinal tissue homeostasis. However, their functionality significantly declines with age, leading to diminished tissue regeneration and an increased risk of age-associated diseases.

**Methods:**

This study investigates the effects of Dapagliflozin (DAPA), a novel insulin sensitizer and SGLT2 inhibitor, on aging ISCs using the *Drosophila melanogaster* model. Our findings demonstrate that DAPA can inhibit the MAPK signaling pathway, as confirmed by network pharmacology analysis and molecular docking experiments.

**Results:**

DAPA ameliorates ISC aging, improves intestinal function (including enhanced fecal excretion, restored intestinal barrier integrity and acid-base balance), and enhances healthspan. These results highlight the potential of DAPA as an anti-aging therapeutic agent.

**Discussion:**

This study provides new evidence for the application of DAPA as an anti-aging treatment.

## 1 Introduction

Aging is a complex biological process characterized by multidimensional and multilevel functional decline across cells, tissues, and organs, thereby increasing the risk of diseases (López-Otín et al., 2013, López-Otín et al., 2023). Normal intestinal stem cells (ISCs) possess the dual abilities of self-renewal and differentiation, which are essential for maintaining tissue and organ homeostasis (Ohlstein and Spradling, 2006). However, their functionality significantly declines with age, resulting in diminished tissue regeneration capacity and an elevated risk of age-related diseases (Brunet, Goodell, and Rando, 2023; Ermolaeva et al., 2018). Consequently, combating ISC aging represents a promising yet challenging field (Du et al., 2021; Yan et al., 2022). Investigating small molecule drugs to mitigate age-related impairments in ISC function can promote healthy aging and complement the mechanisms of age-related decline.

Dapagliflozin (DAPA), a novel insulin sensitizer and SGLT2 inhibitor, exerts direct, sugar-independent effects by reducing oxidative stress and endoplasmic reticulum stress (Shibusawa et al., 2019), restoring mitochondrial health, stimulating mitochondrial biogenesis (Lee et al., 2019), and decreasing pro-inflammatory and profibrotic pathways (Kounatidis et al., 2023). These effects rejuvenate aging cells, tissues, and organs (O'Keefe et al., 2023), and are associated with a reduced risk of various common age-related diseases, including heart failure (Butt et al., 2022), chronic kidney disease (Jhund et al., 2021), atrial fibrillation (Butt et al., 2022), cancer (Basak et al., 2023), gout (Lai et al., 2023), neurodegenerative diseases (Wu et al., 2023), and atherosclerosis (Ortega et al., 2019). Recent Studies have shown that DAPA can reduce the accumulation of reactive oxygen species (ROS) in cells and improve age-related endothelial dysfunction (Tai et al., 2023). Moreover, recent studies have shown that the SGLT2 inhibitors canagliflozin enhances the clearance of senescent cells, thereby improving age-related phenotypic changes and extending lifespan (Katsuumi et al., 2024). We hypothesize that DAPA can modulate ISC function and delay aging, which needs further validation.

*Drosophila melanogaster*, a model organism renowned for aging research, serves as an ideal model for studying ISC aging (Promislow et al., 2022). During homeostasis, the midgut of fly is composed of ISCs and various differentiated cell types, closely resembling the complexity of the mammalian gut (Jasper, 2020). Fly ISCs are characterized by their expression of Delta (Dl, a Notch ligand) and Escargot (Esg, a transcription factor). ISCs divide and differentiate into enteroblasts (EBs) and enteroendocrine progenitor cells (EEPs), which further differentiate into enterocytes (ECs) and enteroendocrine cells (EEs), thereby maintaining the self-renewal of the intestinal epithelium (Biteau et al., 2008; Ayyaz and Jasper, 2013; Biteau et al., 2011). In the aging fly intestine, aberrant ISCs function leads to hyper-proliferation,thereby disrupting intestinal homeostasis and impairing intestinal function. Extensive research has demonstrated that alleviating ISC hyperplasia, which is caused by dysplasia (Rodriguez-Fernandez et al., 2020), plays a crucial role in extending lifespan (Biteau et al., 2010; Du et al., 2020). Thus, the fly intestine is a valuable model for studying the impact and mechanism of DAPA on stem cell aging.

Research has shown that the MAPK signaling pathway exerts regulatory influence over ISC aging in *Drosophila* (He et al., 2020), and modulating MAPK activity can contribute to maintaining normal function (Ureña et al., 2024; Zhang et al., 2019; Park et al., 2009). In our study, using the *Drosophila* gut as a model system, we found that DAPA alleviates hyper-proliferation of ISCs, maintains gut homeostasis, improves age-related declines in intestinal function, and prolongs the lifespan of *Drosophila.* Additionally, our findings indicate that these effects of DAPA are mediated through the inhibition of the MAPK signaling pathway. Therefore, our study not only highlights DAPA’s potential as a novel anti-aging drug but also underscores the importance of the MAPK pathway in stem cell aging and longevity. In conclusion, the study demonstrates that DAPA can serve as an effective and safe anti-aging drug to promote healthy aging.

## 2 Results

### 2.1 Dapagliflozin prevents gut hyperplasia of ISCs in aged *Drosophila melanogaster*

**FIGURE 1:**
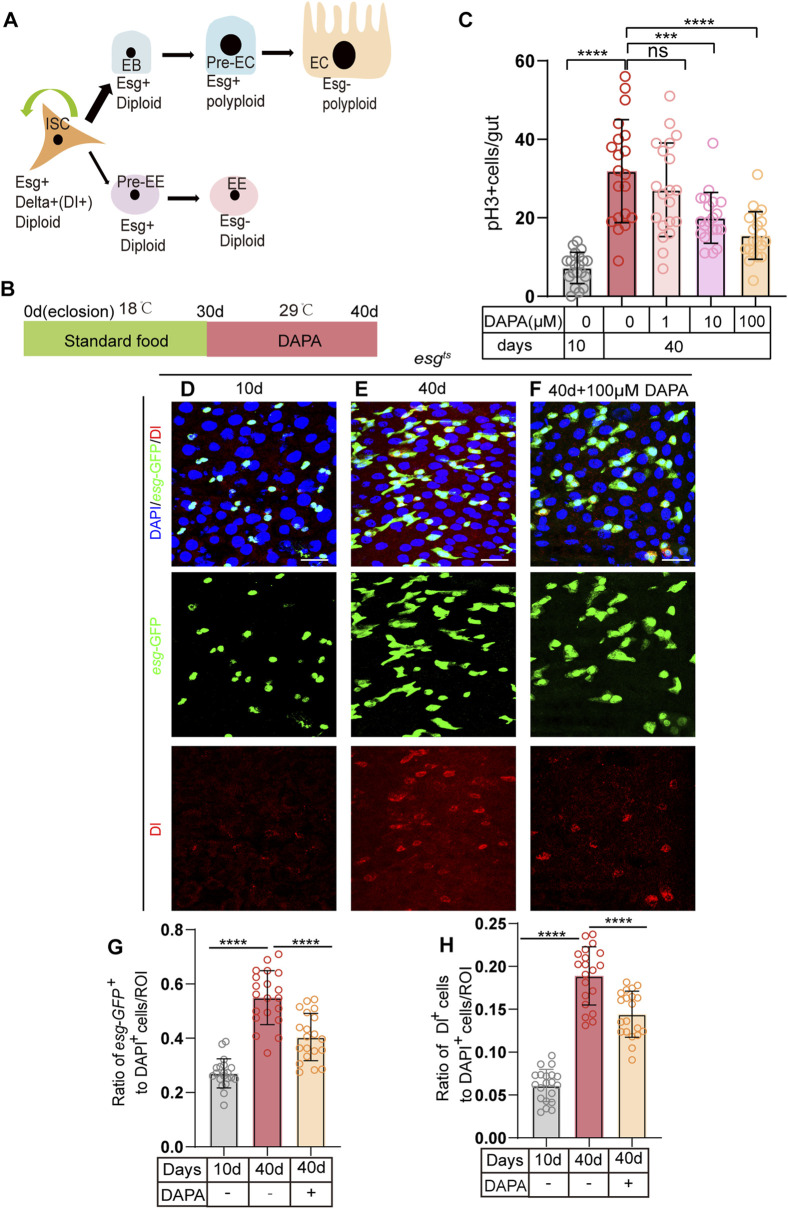
Dapagliflozin Prevents gut hyperplasia of ISCs in Aged *Drosophila melanogaster*. **(A)** The lineages of *Drosophila* intestinal stem cells (ISCs). ISCs (identified by Esg^+^,delta^+^) undergo asymmetric division to self-renew and give rise to enteroblasts (EBs) or enteroendocrine progenitor cells (EEPs/Pre-EE). EBs differentiate to form enterocytes (ECs), while EEPs differentiate to form enteroendocrine cells (EEs). **(B)** Schematic diagram depicting the process of oral DAPA in *Drosophila*. **(C)** The number of pH3+ cells (a marker of mitotic activity) was assessed in the whole guts of 10-day-old and 40-day-old female flies (esg^ts^) without DAPA supplementation and with three concentrations of DAPA (1 μM, 10 μM, and 100 µM). **(D–F)** Immunofluorescence images of posterior midguts of 10- **(D)** and 40-day-old **(E)** female flies without DAPA supplementation and 40-day-old male flies with 100 μM DAPA supplementation **(F)** stained with DAPI (blue; nuclei), GFP (green; ISCs and progenitor cells marker), and Dl (red; ISCs marker). The top panels represent the merged images, the middle panels represent *esg*-GFP, and the bottom panels represent Dl. Scale bars represent 20 µm. **(G)** The ratio of *esg-*GFP^+^ cells to DAPI^+^ cells per ROI (region of interest) in posterior midguts of 10- and 40-day-old female flies without DAPA supplementation and 40-day-old female flies with 100 μM DAPA supplementation. **(H)** The ratio of Dl^+^ cells to DAPI^+^ cells per ROI (region of interest) in female flies experiments. The numbers of counted guts are 20. Data are represented as means ± SD. ANOVA or unpaired Kruskal Wallis test be used, *p < 0.05, **p < 0.01, ***p < 0.001, and ****p < 0.0001, and ns indicates p > 0.05.

In the *Drosophila* midgut, intestinal stem cells (ISCs) maintain intestinal cell homeostasis through proliferation and differentiation (Figure 1A). However, in aging *Drosophila*, the regulation of stem cell proliferation is dysregulated, leading to abnormal accumulation of Esg + cells (including ISCs, EBs, and pre-EE), while the number of fully differentiated intestinal cells decreases, thereby impairing normal intestinal function (Choi et al., 2008).

To investigate whether certain small molecule compounds can prevent age-related ISC dysfunction, we utilized the; esg-Gal4, UAS-GFP, tub-Gal80TS/Cyo; system (hereafter referred to as esgts) to express green fluorescent protein (GFP) under the control of the esg gene at 29°C. This system allowed us to monitor and quantify changes in esg-GFP + cells in real-time, enabling us to screen for small molecule drugs that can alleviate age-related ISC dysfunction. The esgts system is based on the TARGET system, which is a powerful tool for spatiotemporal gene expression targeting in *Drosophila* (McGuire et al., 2004). The TARGET system utilizes a temperature-sensitive GAL80 protein (GAL80ts) to control the activity of GAL4. At the permissive temperature (e.g., 18°C), GAL80ts binds to and inhibits GAL4, preventing it from activating downstream UAS-driven gene expression. When the temperature is shifted to the restrictive temperature (e.g., 29°C), GAL80ts loses its binding affinity for GAL4, allowing GAL4 to activate the expression of genes under the control of UAS elements. This temperature-dependent regulation enables precise temporal control of gene expression, making it an ideal system for studying the effects of small molecule compounds on ISC function over time.

At 30 days old, flies are in the middle and old age stages, and they begin to show physiological changes associated with aging, such as decreased intestinal cell homeostasis, abnormal accumulation of Esg + cells, and a reduction in the number of fully differentiated intestinal cells. We supplemented the diet of 30-day-old flies with Dapagliflozin (DAPA) and maintained them at 29°C for 10 days (Figure 1B). we used the entire gut for the pH3+ cell (phosphorylated histone H3, which specifically stains dividing ISCs) count, which is a common approach in the field to provide a comprehensive assessment of cell proliferation across the entire organ. This method allows us to capture the overall proliferative activity within the gut, which is essential for understanding the biological processes we are investigating. Among the three tested concentrations of DAPA (1 μM, 10 μM, and 100 µM), the 100 µM concentration exhibited the most pronounced effect on pH3+, significantly mitigating intestinal hyperplasia caused by dysplasia in aged flies (Figure 1C). Compared to aged flies without DAPA supplementation, those supplemented with DAPA for 10 days showed a significant reduction in the number of esg + cells (Figures 1D–G). Further quantification of Dl + cells (ISC markers) demonstrated that the number of Dl + cells (Figures 1D–H) in aged flies *a* supplemented with 100 µM DAPA was significantly lower than in the control group. These findings suggest that DAPA supplementation prevents excessive ISC proliferation and subsequent intestinal hyperplasia in aged flies.

### 2.2 Dapagliflozin prevents age-related gut dysfunction in aged *Drosophila melanogaster*

**FIGURE 2:**
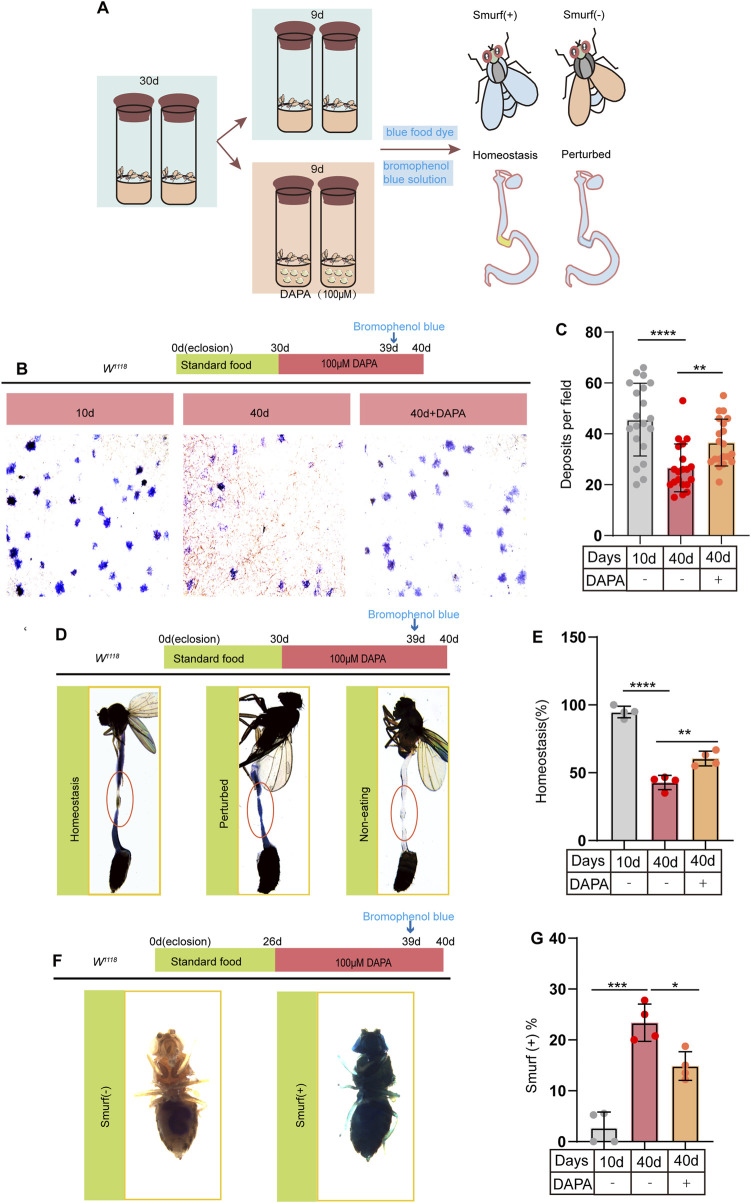
Dapagliflozin Prevents Age-Related Gut Dysfunction in Aged *Drosophila melanogaster* **(A)** Schematic diagram depicting the procedure of DAPA administration in *Drosophila*: *Drosophila* were cultured at 26°C until reaching 30 days of age, following which they were randomly divided into two groups: a control group and a group supplemented with DAPA. After a feeding period of 9 days, flies were selected for “Smurf,” deposits and intestinal “Homeostasis,” respectively. **(B)** Representative images of excretion deposits from female flies at 10 and 40 days old without DAPA supplementation, and 40 days old with 100 µM DAPA supplementation. **(C)** Quantification of excretion deposits from female at 10 and 40 days old without DAPA supplementation, and 40 days old with 100 µM DAPA supplementation. Excretions are quantified for 6 fields in each group of 20 flies. **(D)** Representative images of the intestinal acid-base homeostasis and the non-eating intestine: “Homeostasis” corresponds to CCR as yellow; “Perturbed” corresponds to CCR as blue; “Non-eating” indicates that the flies did not ingest food, and the guts are not stained with bromophenol blue **(E)**. The percentage of “Homeostasis” female in experiment. Each group included 20 flies. **(F)** Representative images of the gut leakage:Smurf (+) corresponds to the leakage of blue dye from the gut into surrounding tissues. **(G)** The ratio of the female flies of smurf (+) in experiment. Each group included 20 flies. Three independent experiments were conducted more than three times. Data are represented as means ± SD. ANOVA or unpaired Kruskal Wallis test be used.*p < 0.05, **p < 0.01, ***p < 0.001, and ****p < 0.0001, and ns indicates p > 0.05.

To further explore the potential effects of DAPA supplementation on intestinal function, we investigated its impact on the gut of aged flies. Previous studies have demonstrated that age-related dysfunction of ISCs leads to a significant deterioration of intestinal function in flies, including disruption of gastrointestinal acid-base homeostasis, reduced fecal excretion, and compromised intestinal barrier integrity (Lemaitre and Miguel-Aliaga, 2013; Kühn et al., 2020). To address this issue, flies were fed a diet with or without DAPA supplementation and subsequently treated with bromophenol blue (BPB) or non-absorbable blue dye (Figure 2A). BPB is a pH indicator. Following BPB feeding, wild-type fly midguts were examined for acidification. A yellow copper cell region (CCR) revealed acidification (pH < 2.3), while the blue anterior and posterior midgut indicated a neutral to basic pH (pH > 4). Our results demonstrated that DAPA supplementation significantly enhanced fecal excretion in aged flies (Figures 2B, C). In flies, the size and function of the CCR decline with age, resulting in disrupted acid-base balance in the gut (Dubreuil, 2004). We observed that DAPA supplementation significantly restored acid-base balance in the gut of aged flies (Figures 2D, E). The integrity of the intestinal barrier is essential for maintaining epithelial homeostasis, defending against pathogens, and promoting immune tolerance to commensal bacteria (Martel et al., 2022). Furthermore, to assess the impact of DAPA on intestinal barrier function, we conducted the blue dye assay. Smurf (+) indicates that the flies exhibit compromised intestinal barrier function, with visible blue dye staining on the body surface due to leakage. Smurf (−) indicates that the intestinal barrier function is intact, with no visible blue dye staining (Figure 2F). Our study demonstrates that supplementation with DAPA significantly enhances intestinal barrier function in aged flies (Figure 2G). In conclusion, these findings demonstrate that DAPA ameliorates age-related intestinal dysfunction in aged flies.

### 2.3 Dapagliflozin extends lifespan under both natural and stress-induced conditions in *Drosophila melanogaster*

**FIGURE 3:**
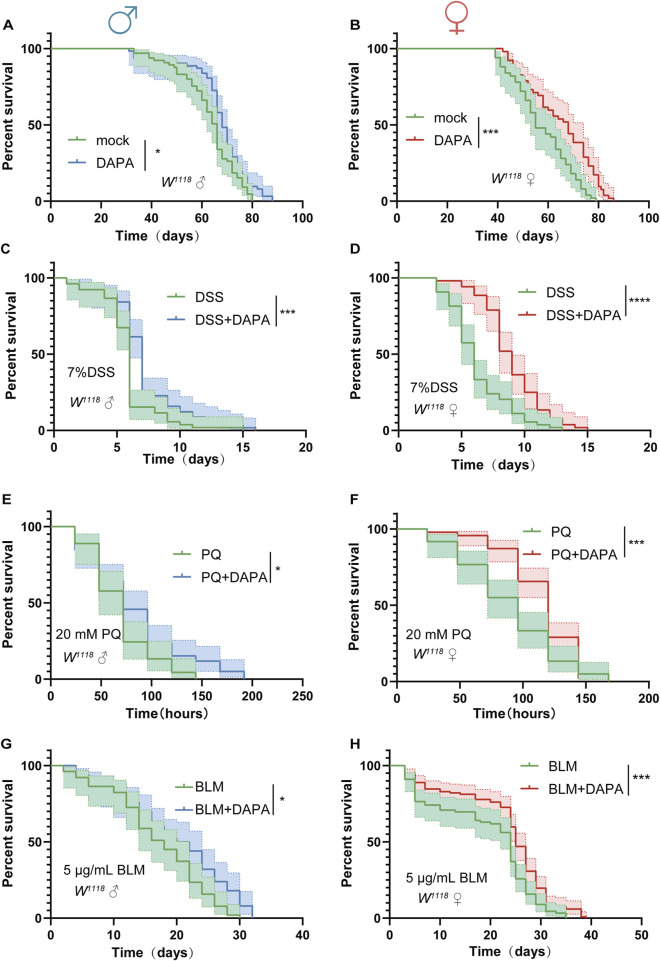
Dapagliflozin Extends Lifespan Under Both Natural and Stress-Induced Conditions in *Drosophila* **(A, B)** Survival percentage of male flies (*W* ^*1118*^, control: n = 65, DAPA: n = 65) **(A)** and female flies (*W* ^*1118*^, control: n = 50, DAPA: n = 50) **(B)** with or without DAPA supplementation. **(C, D)** Survival percentage of male flies (*W* ^*1118*^, control: n = 52, DAPA: n = 57) **(C)** and female flies (*W* ^*1118*^, control: n = 54, DAPA: n = 51) **(D)** with or without DAPA supplementation during 7% dextran sulfate sodium (DSS) treatment. **(E, F)** Survival percentage of male flies (*W* ^*1118*^, control: n = 45, DAPA: n = 57) **(E)** and female flies (*W* ^*1118*^, control: n = 60, DAPA: n = 90). **(F)** with or without DAPA supplementation during 20 mM paraquat (PQ) treatment. **(G, H)** Survival percentage of male flies (*W* ^*1118*^, control: n = 48, DAPA: n = 47) **(G)** and female flies (*W* ^*1118*^, control: n = 89, DAPA: n = 117) **(H)** with or without DAPA supplementation during 5 μg/mL bleomycin (BLM) treatment. Three independent experiments were conducted. Lifespan analysis was performed using the log-rank test, *p < 0.05, **p < 0.01, ***p < 0.001, and ****p < 0.0001, and ns indicates p > 0.05.

Intestinal health is closely related to lifespan extension. Accumulating evidence has demonstrated that maintaining intestinal homeostasis can contribute to longevity (Jing et al., 2025; Choi and Augenlicht, 2024). we observed that DAPA alleviates age-related ISC hyperplasia and prevents age-related gut dysfunction in aged flies, which prompted us to further investigate the effects of DAPA supplementation on the lifespan of flies. Initially, DAPA was supplemented into the diet of the experimental group, and the results demonstrated that DAPA supplementation significantly extended the lifespan of flies compared to the control group (Figures 3A, B).

Subsequently, we sought to explore whether DAPA could extend lifespan under various environmental stress conditions. Flies were exposed to various environmental stress models, including dextran sulfate sodium (DSS)-induced ulcerative colitis (Yan et al., 2023), paraquat (PQ)-induced oxidative damage (Lyles et al., 2021), and bleomycin (BLM)-induced DNA damage (Du et al., 2020). Under DSS-induced stress, we observed that DAPA mitigated the damage to the tight junctions between intestinal cells. Specifically, the damage to the tight junctions, as marked by Armadillo (Arm), was less severe in the DAPA-treated group compared to the control group (Supplementary Figure S1A). In the PQ-induced oxidative stress model, the transcriptional levels of ROS-related genes were downregulated in the DAPA-supplemented group, suggesting that DAPA may enhance antioxidant capacity (Supplementary Figure S1B). Furthermore, in the BLM-induced DNA damage model, the transcriptional levels of DNA damage-related genes were significantly reduced in the DAPA-treated group, indicating that DAPA could mitigate DNA damage (Supplementary Figure S1C). The scavenging effects of DAPA supplementation on oxidative and DNA damage likely involve multiple mechanisms, including direct antioxidant actions, regulation of DNA repair mechanisms, and inhibition of inflammatory responses (Arow et al., 2020; Alsereidi et al., 2024). The synergistic action of these mechanisms may contribute to the extended lifespan of fruit flies under various stress conditions.

Under DSS-induced stress, DAPA significantly extended the lifespan of flies (Figures 3C, D). In the PQ-induced acute aging model, DAPA supplementation significantly extended the lifespan of flies and effectively mitigated acute oxidative stress (Figures 3E, F). Additionally, in the BLM exposure model, DAPA significantly prolonged the lifespan of flies (Figures 3G, H). In summary, our findings demonstrate that DAPA supplementation not only extends the normal lifespan of flies but also significantly prolongs lifespan under various stress-induced model.

### 2.4 Dapagliflozin alleviates aging-induced gut hyperplasia of ISCs primarily through repressing the MAPK signaling pathway

Based on prior studies demonstrating that DAPA may regulate the MAPK signaling pathway (Wang et al., 2022; Phongphithakchai et al., 2025) and our PPI network/pathway enrichment analysis (Supplementary Figure S2), we further explored the potential molecular mechanisms using both PPI network analysis and pathway enrichment analysis (Supplementary Figure S2). These analyses collectively suggest that DAPA may exert its protective effects via the EGFR/MAPK signaling pathway in ISC aging. To test this hypothesis, we conducted the following experiments.

**FIGURE 4:**
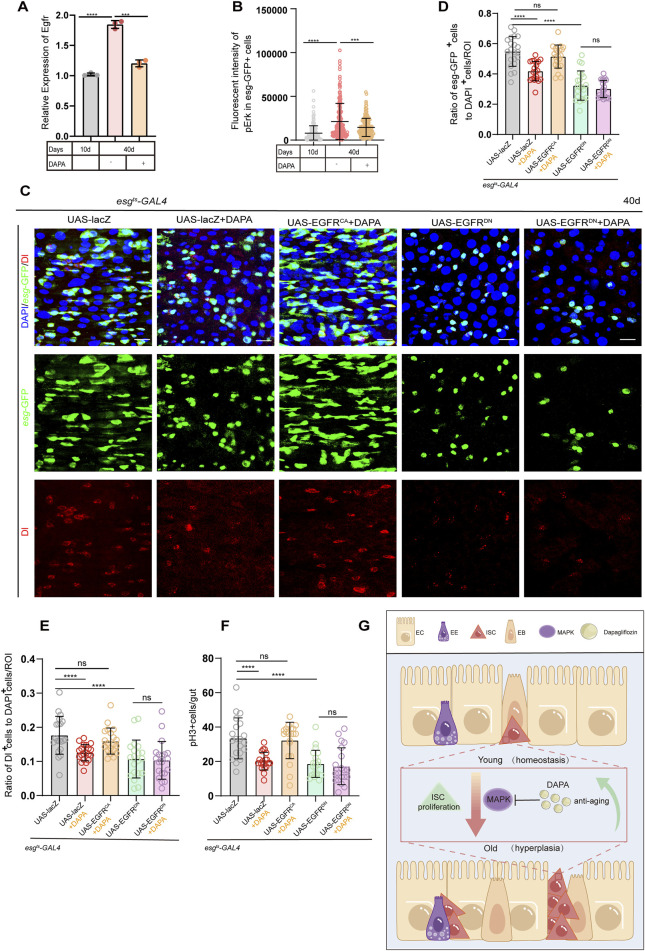
Dapagliflozin alleviates aging-induced gut hyperplasia of ISCs primarily through repressing the MAPK signaling pathway **(A)** RT-qPCR was used to measure the mRNA levels of Egfr gene in esg + ISCs isolated from the midguts of esg-GFP *Drosophila* at three different time points: 10 days, 40 days, and 40 days + DAPA **(B)** Quantification of fluorescent intensity of pErk in *esg*-GFP^+^ cells from 40-day-old female flies (e*sg* ^*ts*^ *-Gal4* > *UAS-lacZ*) at three different time points: 10 days, 40 days, and 40 days + DAPA. Cells were stained with pErk antibody. Each dot represents one esg-GFP + cell. n = 165 per group, with each n representing one esg-GFP + cell, and the n values for each group were derived from 30 flies.**(C)** Immunofluorescence images of posterior midguts of female flies carrying e*sg* ^*ts*^ *-Gal4*-driven *UAS-lacZ*, *UAS-lacZ* + DAPA*, UAS-EGFR* ^*CA*^ + DAPA, *UAS-EGFR* ^*DN*^, and *UAS-EGFR* ^*DN*^ + DAPA stained with DI,DAPI and GFP. The top panels represent the merged images, the middle panels represent esg-GFP, and the bottom panels represent Delta (Dl). Scale bars represent 20 µm. **(D)** The ratio of *esg-*GFP^+^ cells to DAPI^+^ cells per ROI (region of interest) in posterior midguts of female flies in experiment. **(E)** The ratio of Dl^+^ cells to DAPI^+^ cells per ROI (region of interest) in posterior midguts of female flies. **(F)** The number of pH3^+^ cells in the whole guts of female flies carrying. **(G)** Model of mechanism: how DAPA modulates ISC aging. In *Drosophila melanogaster*, DAPA inhibits the MAPK signaling pathway, thereby suppressing intestinal hyperplasia during aging. Data are represented as means ± SD. ANOVA or unpaired Kruskal Wallis test be used, *p < 0.05, **p < 0.01, ***p < 0.001, and ****p < 0.0001, and ns indicates p > 0.05.

The Epidermal Growth Factor Receptor (EGFR) is a well-known regulator of ISC activity. Activation of EGFR leads to the phosphorylation and activation of ERK, which in turn promotes ISC proliferation and maintains intestinal homeostasis. This pathway directly feeds into the MAPK cascade, making it a primary upstream regulator (Zhang et al., 2022; Li et al., 2015). To further explore the potential of DAPA in regulating the EGFR signaling pathway, we first measured the transcriptional level of EGFR in ISCs and found that DAPA supplementation significantly reduced EGFR transcription in intestinal ISCs (Figure 4A). Subsequently, we observed that the fluoreoxidative stress and endoplasmicscence intensity of phosphorylated Extracellular Signal-Regulated Kinase (pERK), a marker of MAPK activation, was also significantly reduced in the DAPA supplementation group, indicating that DAPA alleviated intestinal hyperplasia by inhibiting pERK expression (Figure 4B; Supplementary Figure S3A).

To further confirm the role of DAPA in regulating the EGFR signaling pathway, we first induced overexpression of the dominant-negative form of EGFR (EGFR^DN^) in *Drosophila*. We hypothesized that DAPA slows the aging of *Drosophila* ISCs by inhibiting the EGFR pathway and predicted that no synergistic effect would be observed in *Drosophila* expressing EGFR^DN^ (driven by esgts-gal4) upon DAPA administration. Compared to normal aged *Drosophila*, the number of esg-GFP + cells was significantly suppressed in aged *Drosophila* with esgts-gal4-driven EGFR^DN^. Consistent with our expectations, DAPA supplementation did not lead to a further reduction in esg-GFP + cells (Figures 4C, D), and statistical analysis demonstrated similar effects in Dl+ and pH3+ cell counts (Figures 4E, F; Supplementary Figure S3B).

In addition, we conducted experiments with the constitutively active form of EGFR (EGFR^CA^). We hypothesized that overexpression of EGFR^CA^ would counteract the protective effects of DAPA on ISC dysfunction. The results showed that, compared to normal aged *Drosophila*, the number of esg-GFP + cells was significantly increased in *Drosophila* expressing EGFR^CA^ (Figures 4C, D), indicating that EGFR^CA^ overexpression induces ISC hyperproliferation. Notably, DAPA treatment failed to significantly reduce the number of esg-GFP + cells in *Drosophila* expressing EGFR^CA^ (Figures 4C, D), with parallel resistance observed in Dl+ and pH3+ cell counts (Figures 4E, F; Supplementary Figure S3B). This further substantiates that EGFR^CA^ overexpression counteracted the protective effects of DAPA (Figures 4C–F). These findings suggest that DAPA-induced mitigation of age-related ISC hyperproliferation in *Drosophila* is associated with inhibition of the MAPK/EGFR signaling pathway (Figure 4G).

## 3 Discussion

Researchers in the field of aging have long been dedicated to understanding the mechanisms of aging and identifying precise therapeutic strategies to delay aging and alleviate age-related diseases. A critical frontier in biomedical science is the pursuit of interventions that can mitigate the adverse effects of aging on stem cell function. This study demonstrates for the first time that Dapagliflozin (DAPA) ameliorates intestinal stem cell (ISC) aging in *Drosophila melanogaster* by repressing the MAPK signaling pathway. Our findings reveal that DAPA supplementation reduces age-related ISC hyperplasia (evidenced by decreased pH3+ and Dl + cells), restores intestinal barrier integrity, acid-base homeostasis, and excretory function, while extending lifespan under both natural and stress-induced conditions. Network pharmacology and molecular docking analyses further validate that DAPA suppresses ERK phosphorylation by targeting EGFR/MAPK3, thereby maintaining ISC homeostasis. These results provide direct evidence for DAPA as a potential anti-aging therapeutic agent.

The extracellular-signal-regulated kinases (ERK) signaling pathway, a classic pathway of mitogen-activated protein kinases (MAPK), plays a crucial role in regulating cell growth, differentiation, survival, inflammation (Fang and Richardson, 2005; Chen et al., 2020; Schafer et al., 2017). Numerous studies have demonstrated a negative correlation between ERK and healthy lifespan, and a decrease in ERK is able to inhibit age-related degenerative diseases (Tarragó et al., 2018; Sun et al., 2020). Additionally, as one of the most important pathways regulating cell proliferation, the overactivation of the ERK pathway can autonomously stimulate the proliferation of intestinal stem cells (Zhang et al., 2019). Live-cell imaging of colon monolayers reveals that ERK is localized in the stem cell niche to maintain epithelial homeostasis (Pond et al., 2022). Furthermore, in *Drosophila* intestine, ERK regulates the homeostasis and regeneration of intestinal stem cells (Jiang et al., 2011).

Beyond its glucose-lowering effects, DAPA has been shown to exert a range of beneficial effects unrelated to blood glucose control. Consistent with previous studies, SGLT2 inhibitors (including DAPA) exert anti-aging effects through multiple pathways, such as mitigating oxidative stress and mitochondrial dysfunction (Tai et al., 2023; Jiang et al., 2023; Katsuumi et al., 2024). However, their role in intestinal stem cell aging remains underexplored. Our work bridges this gap by uncovering DAPA’s unique mechanism via MAPK pathway inhibition. Notably, hyperactivation of MAPK signaling is a key driver of age-related ISC dysfunction, and DAPA’s suppression of this pathway aligns with the pro-aging role of ERK signaling in mammals (Ureña et al., 2024; Nászai et al., 2021; Kabiri et al., 2018).

The anti-aging effects of Dapagliflozin (DAPA) on intestinal stem cell (ISC) dysfunction may involve both SGLT2-dependent and -independent mechanisms. While DAPA is primarily known for its glycemic control through SGLT2 inhibition, recent studies suggest that its benefits on ISC function could be mediated by non-glycemic actions. These include anti-inflammatory and antioxidant effects, which reduce oxidative stress and inflammation via pathways such as NOX4/p38MAPK inhibition (Dihoum et al., 2024; Alsereidi et al., 2024); autophagy regulation, which supports cellular homeostasis by clearing damaged organelles and proteins n (Jaikumkao et al., 2021; Arab et al., 2021); and cardiovascular protection through pathways like ALDH2 and SK2 modulation (James et al., 2024; Madero et al., 2024; Panico et al., 2024). DAPA is known to inhibit the ERK signaling pathway, thereby mitigating age-related dysfunction of ISC. However, DAPA may also exert its effects by modulating other MAPK pathways, such as JNK and p38 MAPK (Sun et al., 2019). For instance, in models of diabetic cardiomyopathy, DAPA has been shown to provide direct cardioprotection by modulating the NHE1/MAPK signaling pathway (Lin et al., 2022). Moreover, DAPA has been reported to attenuate cellular stress and inflammation through the modulation of the PI3K/AKT pathway (Alsereidi et al., 2024). Although there is currently no direct evidence demonstrating DAPA’s effects on JNK and p38 MAPK signaling pathways in our *Drosophila* models, considering DAPA’s regulatory actions on these pathways in other systems, we cannot rule out the possibility that it may also act through these pathways in *Drosophila*. Although these multifaceted actions highlight the potential for non-glycemic mechanisms in DAPA’s anti-aging effects, further research is needed to elucidate the precise contributions of SGLT2-dependent and -independent pathways using *Drosophila* diabetes models (Miao et al., 2022). This will contribute to a more comprehensive understanding of DAPA’s anti-aging potential and provide a theoretical basis for the development of novel anti-aging therapeutics.

While this study establishes DAPA’s efficacy in flies, further validation in mammalian models is essential. Additionally, whether DAPA’s inhibition of MAPK synergizes with other aging-related pathways (e.g., AMPK or mTOR) warrants investigation (Sung et al., 2024). Future research should also address dose-response relationships and long-term safety to facilitate clinical translation. In conclusion, our findings position DAPA as a promising anti-aging compound and underscore the therapeutic potential of targeting MAPK signaling to combat stem cell aging.

## 4 Materials and methods

### 4.1 *Drosophila* strains and culture

The following *Drosophila* strains were used in this study: *w* ^*1118*^ line (BDSC# 3605), *UAS-lacZ* line (from Allan Spradling), *esg* ^*ts*^ *-Gal4* line (from Benjamin Ohlstein), *UAS-EGFR* ^*CA*^ line (BDSC# 9533) and *UAS-EGFR* ^*DN*^ line (BDSC #5364).

Flies were maintained on standard cornmeal-agar medium (80 g sucrose, 50 g cornmeal, 20 g glucose, 18.75 g yeast, 5 g agar, and 30 mL propionic acid per 1 L of water) and kept at standard conditions (25°C, 60% relative humidity, and 12 h light/dark cycle). Unless indicated otherwise, only mated females were used in this study (due to the higher incidence of age-related intestinal dysfunction observed in female fruit flies). The gene overexpression or knockdown mediated by the esgts-Gal4 *Drosophila* line was repressed at 18°C and activated at 29°C.

### 4.2 Drugs treatment

#### 4.2.1 Dapagliflozin treatment

Dapagliflozin (Macklin, Shanghai, China, #D830111) was first dissolved in deionized water, then added to standard food to obtain different concentrations. Flies were collected randomly and maintained in the medium with different concentrations of Dapagliflozin (DAPA).

#### 4.2.2 Bleomycin and paraquat treatment

A chromatography paper was cut into 3.7 × 5.8 cm strips and soaked in 25 μg/mL BLM (Aladdin, B107423), or 20 mM PQ (Aladdin, M106761) dissolved in 5% (wt/vol) sucrose. After being starved for 1 h, flies were transferred into vials with the BLM or PQ solution–saturated chromatography paper with 5% sucrose.

### 4.3 Immunofluorescence

*Drosophila* intestines were dissected in cold phosphate-buffered saline (PBS), fixed at room temperature for 30 min with 4% paraformaldehyde (PFA) (for anti-dpErk immunostaining, guts were fixed in 8% paraformaldehyde for 50 min), and then washed three times (10 min each) in PBS containing 0.1% Tween-20 (PBST). Tissues were immersed in the primary antibodies diluted in PBST and incubated overnight at 4°C.

The following primary antibodies were used: chicken anti-GFP, Abcam, 1:1,000; mouse anti-Delta, DSHB, 1:50; rabbit anti phospho-Histone H3 (Ser10), Millipore, 1:1,000; rabbit anti-dpERK, Cell Signaling, 1:500; Mouse anti-Armadillo 1:100, DSHB, #AB_528089,1:100. After washing, guts were incubated with secondary antibodies (Alexa 488, 568 or 647, Invitrogen, 1:2000) and DAPI (Sigma, 1 μg/mL) for more than 2 h at room temperature with shaking. Finally, seal and preserve the intestines by soaking them with the anti-fluorescence quenching agent and placing them on slides.

Immunofluorescence images were captured with a Leica TCS-SP8 confocal microscope and assembled with Application Suite X, Adobe Illustrator, and ImageJ software.

### 4.4 Smurf assay

To test the integrity of the *Drosophila* intestinal barrier (Rera et al., 2011), *Drosophila* treated with different drugs (fed with or without DAPA) were starved for 1 h and then cultured in the medium with added 2.5% (wt/vol) blue food dye (Spectrum Chemical Manufacturing Corp, Shanghai, China, #FD110) for 12 h. Flies leaking blue dye outside the intestine were considered as “Smurf (+)” flies. Smurf (+) indicates that the flies exhibit compromised intestinal barrier function, with visible blue dye staining on the body surface due to leakage. Smurf (−) indicates that the intestinal barrier function is intact, with no visible blue dye staining.

### 4.5 Bromophenol blue assay

To determine the pH of *Drosophila* midguts, the bromophenol blue assay was performed in the following steps (Li et al., 2016): add 200 µL of 2% bromophenol blue solution (Sigma, #B5525, dissolved in 5% sucrose) to the surface of the standard medium, followed by punching several holes with a pipet tip to allow the solution to be fully absorbed. The flies were starved for 1 h and cultured in the above food for 24 h. Then dissect the intestines and capture the images immediately to prevent carbon dioxide from affecting the rendering results.

To ensure accurate assessment of intestinal function, only flies that had consumed food, as indicated by blue staining in their guts (due to bromophenol blue), were included in the analysis. Flies that did not consume food, evidenced by the absence of blue staining, were excluded. The percentage of flies with blue-stained guts, representing those with functional food intake, was then calculated. We refer to flies whose guts were all dyed blue after ingesting food containing bromophenol blue as “perturbed” flies, while whose CCR areas retained yellow were described as “homeostasis.”

### 4.6 *Drosophila* excretion assay

To measure the excretory function of the *Drosophila* intestinal tract [3](Cognigni et al., 2011), *Drosophila* treated with different drugs (fed with or without DAPA) were starved for 1 h and cultured in the bromophenol blue food vial (whose wall was surrounded by chromatography paper) for 24 h. Finally, image the deposits on the paper with a Leica M205 FA stereomicroscope and quantify the number of deposits.

### 4.7 Lifespan assay

To determine the effect of DAPA on the lifespan, 50–100 female or male flies hatched within 48 h were collected and divided equally into five vials, in which the food was mixed with or without DAPA.

To determine the lifespan under stressful conditions, the flies were randomized into the following groups: DSS (7%) + water, DSS (7%) + DAPA (100 µM). PQ (20 mM) + water, PQ (20 mM) + DAPA (100 µM), BLM (5 μg/mL) +water, and BLM (5 μg/mL) + DAPA (100 µM) group. For each group, 50–100 female or male flies hatched within 48 h were collected and divided equally into five vials.

In addition, the female flies used in the experiments are virgins [to avoid confounding factors introduced by mating and to focus on the intrinsic aging processes (Liu et al., 2024)]. The number of dead flies was recorded every 2 days, and the experiments were repeated at least three times.

### 4.8 Network pharmacology

The targets corresponding to DAPA (https://pubchem.ncbi.nlm.nih.gov/) were obtained from the PharmMapper database and filtered the targets with Norm Fit >0.5 (http://www.lilab-ecust.cn/pharmmapper/). The targets of aging were searched by the OMIM (https://omim.org/), TTD (http://db.idrblab.net/ttd/), DrugBank (https://go.drugbank.com/), GeneCards (http://www.genecards.org/), and DisGeNET (https://www.disgenet.org/) databases using “Intestinal stem cells aging” as the keyword. The gene names of these targets were obtained from the Uniprot database after removing the duplicates (http://www.uniprot.org).

The common targets of DAPA and aging were analyzed by the Venny 2.1.0 database to predict the potential targets of DAPA against aging. These common targets were imported into the STRING database (https://cn.string-db.org/), the minimum required interaction score was set to 0.7 and the isolated targets were removed to obtain the protein-protein interaction (PPI) network. Kyoto Encyclopedia of Genes and Genomes (KEGG, https://www.kegg.jp/kegg/pathway.html) enrichment analysis of the common targets was performed using the Metascape database (https://metascape.org/). The enrichment results of KEGG pathways were visualized by the Bioinformatics platform (https://www.bioinformatics.com.cn/).

### 4.9 Molecular docking

The 3D structure of DAPA in the SDF file was obtained from the PubChem database (https://pubchem.ncbi.nlm.nih.gov/), converted to a PDB file via Open Babel 2.4.1 software, used AutoDockTools-1.5.6 to add hydrogen bonds, detect the root, and set rotatable bonds, then saved as PDBQT format. The conformation of proteins obtained from the PDB database were exported to the PDB file, then charged into AutoDockTools-1.5.6 software to remove the water molecules and excess inactive ligands, get hydrogenated and exported to PDBQT format. The molecular docking was performed using AutoDockTools-1.5.6 software and the results were visualized by PyMol (DeLano Scientific, United States) and proteins. plus website (https://proteins.plus/).

### 4.10 Flow cytometry-based isolation of esg^+^ cells from *Drosophila* midguts

A total of 200 female *Drosophila* midgut were dissected under ice-cold PBS (pH 7.4) supplemented with 1% penicillin-streptomycin. The midguts (R1–R5 regions) were carefully excised and immersed in PBS containing diethylpyrocarbonate (DEPC) to inactivate ribonucleases. Tissues were then treated with 1 mg/mL Elastase solution (Sigma, cat. no. E0258) at 25°C for 30 min with periodic gentle mechanical disruption every 10 min to ensure complete dissociation. The resulting cell suspension was passed through a 40 μm cell strainer (Biologix) to generate a single-cell population, which was subsequently sorted using a BD FACS AriaTM III sorter (BD Biosciences) equipped with a 488 nm laser and a 530/30 nm emission filter. Sorting gates were set based on GFP fluorescence intensity and forward/side scatter profiles to exclude esg-cells. Sorted cells were pelleted by centrifugation at 400 × g for 20 min at 4°C, resuspended in 0.2 mL ice-cold PBS/DEPC, and stored at −80°C until further analysis. For each of three biological replicates, approximately 1 × 10^5^–1 × 10^6^ GFP-positive cells were obtained.

### 4.11 RNA isolation and RT-qPCR for *Drosophila*

40 adult midguts or 1 × 10^5^–1 × 10^6^ esg^+^ cells collected into 4°C diethylpyrocarbonate (DEPC)-treated water-PBS solution. Samples were homogenized in RNA-easy Isolation Reagent (Vazyme, R701) for total RNA isolation and cDNA synthesis. RT-qPCRwas performedon aCFX96TouchDeepWell (Bio Rad) usingChamQUniversal SYBRqPCRMasterMix (Vazyme, Q711). The reference standardgroup was Rp49. The expression levels were counted by the2^−△△CT^method.The primers used were listed as below:

Rp49-F:GCCCAAGGGTATCGACAACA.

Rp49-R:GCGCTTGTTCGATCCGTAAC.

Egfr-F: CGACCGTACTACGACGACAGTA.

Egfr-R:TGATCTTGGTGAGGACGATGA.

dmp53-F:CGTGATTGCTGTGGTTACGTGTACT.

dmp53-R:GCTGCAGAATGCGTTGCTGAAATGTG.

chk2-F:ATGGTGCCGTTGTTGATGTGCAGAT.

chk2-R:TGCAGATGTGCGTTGATGTGGTGCCAT.

sod-F: CAAGGGCACGGTTTTCTTC.

sod-R: CCTCACCGGAGACCTTCAC.

cat-F:TTCCTGTGGGCAAAATGGTG.

cat-R:ATCTTCACCTTGTACGGGCA.

### 4.12 Statistical analyses

For all the experiments, the data were processed using GraphPad Prism version 8.0 and presented as average ±SD from at least three independent experiments. Statistical significance was determined using the two-tailed Student’s t-test unless otherwise specified in the figure legends. For all the tests, p < 0.05 was considered to indicate statistical significance.

## Data Availability

The original contributions presented in the study are included in the article/Supplementary Material, further inquiries can be directed to the corresponding authors.

## Supplementary material

SUPPLEMENTARY FIGURE S1 DAPA mitigates oxidative stress and DNA damage in *Drosophila* midgut under different stress conditions **(A)** Reduced Armadillo (Arm) fluorescence intensity in DAPA-treated flies under 7% DSS-mediated intestinal injury.(scale bar = 20 μm). **(B)** Downregulation of ROS-related genes (*sod* and *cat*) in DAPA-treated flies exposed to paraquat (PQ). Gene expression was analyzed by qRT-PCR using RNA extracted from whole midgut tissues (n = 40 guts per group). **(C)** Downregulation of DNA damage-related genes (*chk2* and *dmp53*) in DAPA-treated flies receiving bleomycin (BLM). Data are represented as means ± SD. ANOVA or unpaired Kruskal Wallis test be used, *p < 0.05, **p < 0.01, ***p < 0.001, and ****p < 0.0001, and ns indicates p > 0.05.

SUPPLEMENTARY FIGURE S2 Network pharmacology analysis and molecular docking of Dapagliflozin. **(A)** The structure of DAPA. **(B)** Venn diagram illustrating the intersection between DAPA and Aging. **(C)** PPI network of DAPA and aging intersection targets. **(D)** Kyoto Encyclopedia of Genes and Genomes (KEGG) pathways bubble diagram. **(E)** The gene ontology (GO) functional enrichment analysis revealed a significant enrichment of MAPK-related proteins in biological processes and molecular functions. **(F, G)** Diagram of the molecular docking of DAPA with EGFR or MAPK3, the binding energies for the targets docked into the DAPA are EGFR (−5.44 kcal/mol), MAPK3/ERK (−4.92 kcal/mol).

SUPPLEMENTARY FIGURE S3 Representative images of pErk and pH3 staining in gut of ISCs **(A)** Representative immunofluorescence images of pErk in esg-GFP+ cells from female flies (esgts-Gal4 > UAS-lacZ) at three different time points: 10 days old (10d), 40 days old (40d), and 40 days old with DAPA treatment (40d + DAPA). Cells were stained with pErk antibody. Scale bar represents 20 µm. **(B)** Representative immunofluorescence images of pH3 in the posterior midguts from female flies carrying e*sg* ^*ts*^ *-Gal4*-driven *UAS-lacZ*, *UAS-lacZ* +DAPA*, UAS-EGFR* ^*CA*^ + DAPA, *UAS-EGFR* ^*DN*^, and *UAS-EGFR* ^*DN*^ + DAPA stained with pH3 (red), DAPI (blue). Scale bar represents 20 µm.
