# Supplementary figures and images for: Dapagliflozin ameliorates intestinal stem cell aging by regulating the MAPK signaling pathway in Drosophila
Source: Front Cell Dev Biol. 2025 Apr 23;13:1576258. doi: 10.3389/fcell.2025.1576258 (PMC12055793; doi:10.3389/fcell.2025.1576258)

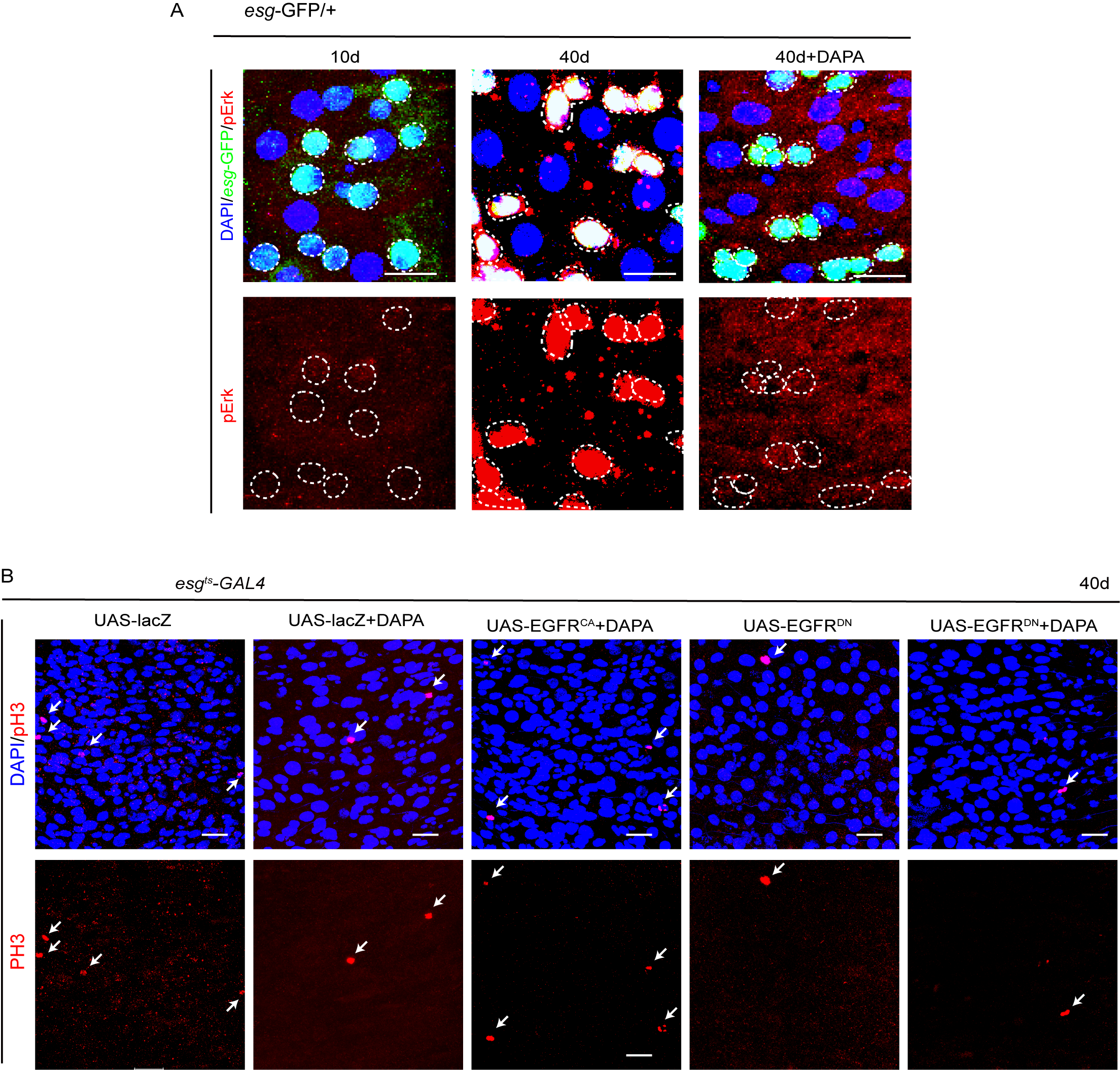

Supplement: Supplementary file 1 [file Image3.tif]

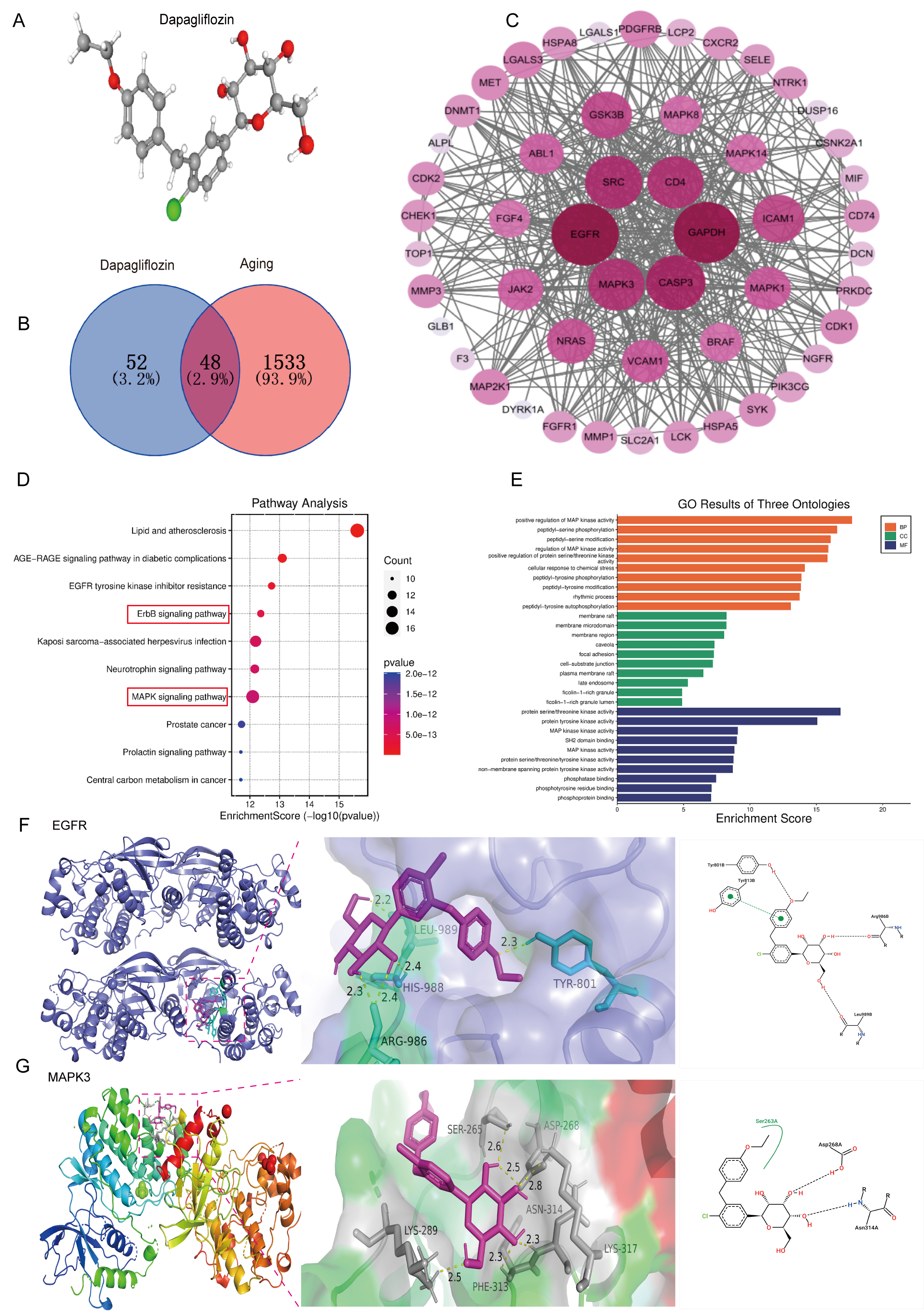

Supplement: Supplementary file 2 [file Image2.tif]

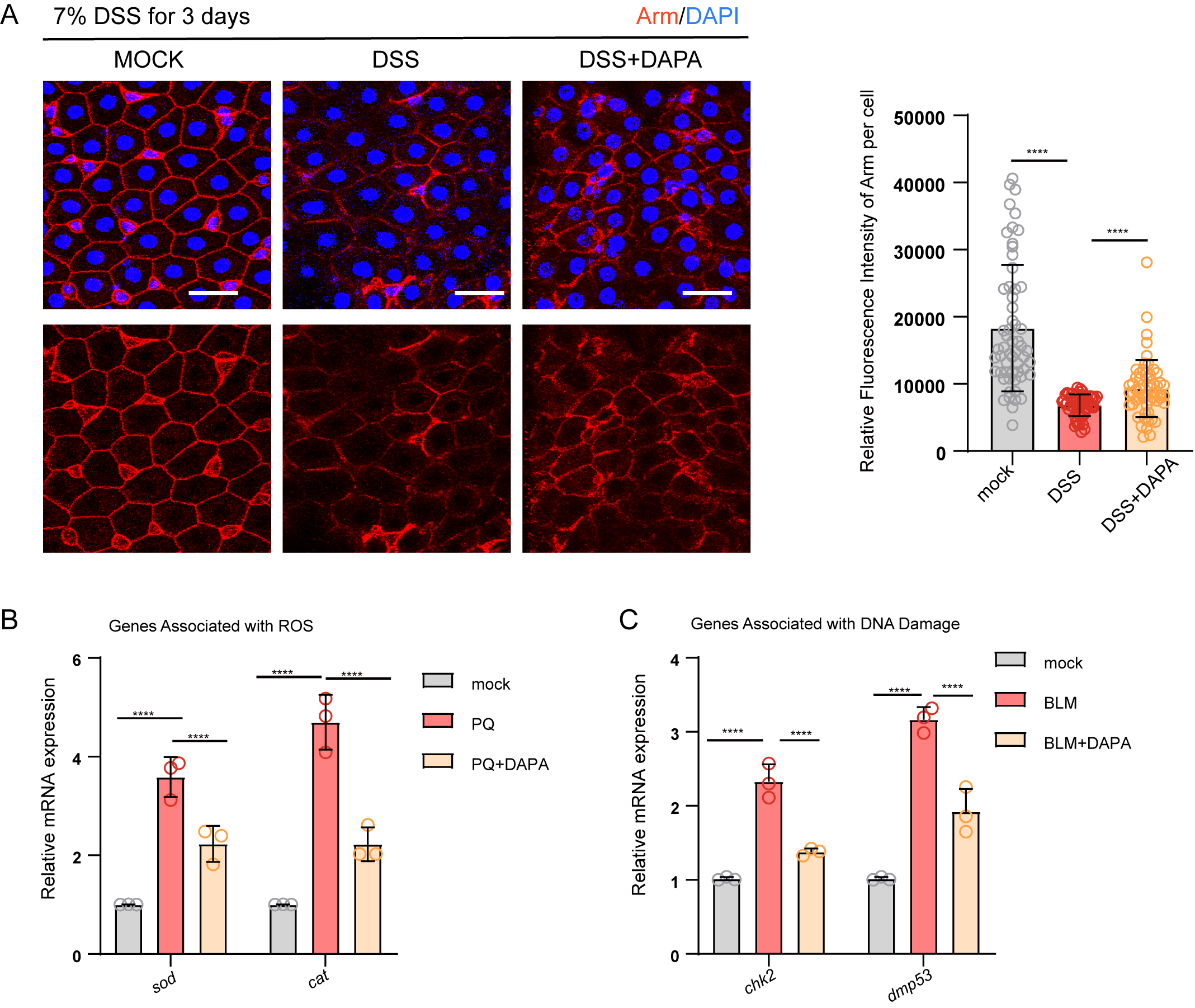

Supplement: Supplementary file 3 [file Image1.tif]
